# Extreme Cardiovascular Risk and LDL-C Target Achievement in People With HIV

**DOI:** 10.1093/ofid/ofag398

**Published:** 2026-07-06

**Authors:** Andrea Giacomelli, Anna Lisa Ridolfo, Letizia Oreni, Giorgia Carrozzo, Maria Vittoria Cossu, Davide Moschese, Giacomo Pozza, Valentina Iannone, Andrea Poloni, Aldo Pietro Maggioni, Andrea Gori, Cristina Gervasoni

**Affiliations:** Department of Infectious Diseases, ASST Fatebenefratelli Sacco, Luigi Sacco Hospital, Milan, Italy; Department of Biomedical and Clinical Sciences, Università degli Studi di Milano, Milan, Italy; Department of Infectious Diseases, ASST Fatebenefratelli Sacco, Luigi Sacco Hospital, Milan, Italy; Department of Infectious Diseases, ASST Fatebenefratelli Sacco, Luigi Sacco Hospital, Milan, Italy; Department of Infectious Diseases, ASST Fatebenefratelli Sacco, Luigi Sacco Hospital, Milan, Italy; Department of Infectious Diseases, ASST Fatebenefratelli Sacco, Luigi Sacco Hospital, Milan, Italy; Department of Infectious Diseases, ASST Fatebenefratelli Sacco, Luigi Sacco Hospital, Milan, Italy; Department of Infectious Diseases, ASST Fatebenefratelli Sacco, Luigi Sacco Hospital, Milan, Italy; Department of Infectious Diseases, ASST Fatebenefratelli Sacco, Luigi Sacco Hospital, Milan, Italy; Department of Infectious Diseases, ASST Fatebenefratelli Sacco, Luigi Sacco Hospital, Milan, Italy; ANMCO Research Center, Heart Care Foundation, Florence, Italy; Department of Infectious Diseases, ASST Fatebenefratelli Sacco, Luigi Sacco Hospital, Milan, Italy; Department of Biomedical and Clinical Sciences, Università degli Studi di Milano, Milan, Italy; Centre for Multidisciplinary Research in Health Science (MACH), Università degli Studi di Milano, Milan, Italy; Department of Infectious Diseases, ASST Fatebenefratelli Sacco, Luigi Sacco Hospital, Milan, Italy

**Keywords:** cardiovascular risk, guidelines, HIV, LDL, target achievement

## Abstract

A substantial proportion of people with HIV and established atherosclerotic cardiovascular disease (ASCVD) meet criteria for extreme cardiovascular risk, yet low-density lipoprotein cholesterol (LDL-C) target achievement remains markedly suboptimal. Bridging the gap between updated guideline recommendations and real-world practice is essential to reduce recurrent ASCVD in this high-risk population.

Atherosclerotic cardiovascular disease (ASCVD) is a leading cause of morbidity and mortality among people with HIV (PWH), despite sustained viral suppression and effective antiretroviral therapy. As PWH age, the burden of ASCVD continues to increase, driven by the combined effects of traditional cardiovascular risk factors and the persistent immune activation and inflammation associated with chronic HIV-1 infection [1, 2]. These mechanisms are not adequately captured by conventional cardiovascular risk prediction tools, which may therefore underestimate ASCVD risk in PWH [3].

The REPRIEVE trial demonstrated that pitavastatin significantly reduced the risk of major adverse cardiovascular events in PWH receiving antiretroviral therapy, including individuals traditionally classified as having low-to-moderate cardiovascular risk [4]. These findings reinforce the need for broader and earlier implementation of cardiovascular prevention strategies in PWH. This issue is particularly relevant in secondary prevention, as longer survival on effective ART has led to an expanding population of PWH living with established ASCVD.

Low-density lipoprotein cholesterol (LDL-C) plays a central causal role in atherosclerosis and represents a key modifiable determinant of ASCVD risk [5]. Robust evidence supports intensive LDL-C lowering for secondary prevention, with progressively lower targets associated with greater reductions in recurrent cardiovascular events [5].

The 2025 Focused Update of the European Society of Cardiology/European Atherosclerosis Society (ESC/EAS) dyslipidemia guidelines has refined cardiovascular risk stratification by introducing an “extreme cardiovascular risk” category among individuals with established ASCVD [6]. This category includes individuals with established ASCVD, including those with a history of myocardial infarction, ischemic stroke, symptomatic peripheral artery disease, or prior coronary, peripheral, or carotid revascularization, who either (i) experience recurrent vascular events despite maximally tolerated statin therapy, or (ii) present with extensive multivessel arterial disease involving at least two distinct arterial territories (coronary, cerebral, or peripheral). For these individuals, an intensified LDL-C target of <40 mg/dL (1.0 mmol/L) is recommended [6].

Although this classification was derived from general population data and has not yet been specifically validated in PWH, it may be particularly relevant in this population given their excess ASCVD risk and higher burden of recurrent cardiovascular events [7, 8]. However, real-world data on the prevalence of extreme cardiovascular risk and LDL-C target achievement among PWH are currently scarce.

The aim of this study was to assess, in a real-world cohort of PWH, the proportion of individuals meeting criteria for extreme cardiovascular risk and to evaluate achievement of guideline-recommended LDL-C targets.

## MATERIALS AND METHODS

We conducted a retrospective observational study at the outpatient HIV clinic of Luigi Sacco Hospital, Milan, Italy. Clinical data were extracted from electronic medical records for all adults attending at least one follow-up visit between June and December 2025.

Demographic characteristics, HIV-related variables (including duration of HIV infection, antiretroviral therapy, and history of AIDS-defining events), cardiovascular risk factors (including hypertension, diabetes mellitus, chronic kidney disease, and smoking status), history of ASCVD, lipid profiles, and lipid-lowering therapies were collected. Individuals with established ASCVD were classified as being at very high or extreme cardiovascular risk according to the 2025 ESC/EAS dyslipidemia guidelines [6]. Established ASCVD (corresponding to very high cardiovascular risk) was defined as a documented history of myocardial infarction, ischemic stroke, symptomatic peripheral artery disease, or previous coronary, peripheral, or carotid revascularization. Extreme cardiovascular risk was defined as established ASCVD plus either recurrent vascular events despite lipid-lowering therapy or extensive multivessel arterial disease involving at least 2 arterial territories (coronary, cerebral, or peripheral) [6].

LDL-C was defined as the last available fasting measurement recorded in the dataset. According to guidelines LDL-C targets were <55 mg/dL for very high-risk individuals and <40 mg/dL for those at extreme risk [6]. Lipid-lowering therapy was categorized as statin or ezetimibe monotherapy, statin plus ezetimibe combination therapy, or therapy with additional LDL-C–lowering agents, including proprotein convertase subtilisin/kexin type 9 (PCSK9) inhibitors and bempedoic acid. People with HIV were considered on stable lipid-lowering therapy if treated for ≥6 months at the time of their most recent lipid assessment.

Descriptive statistics included absolute numbers and percentages for categorical variables and median values with interquartile ranges (IQRs) for continuous variables. The chi-squared test was used to compare proportions between individuals at very high and extreme cardiovascular risk, while the Kruskal–Wallis test was used for continuous variables. No formal sample size calculation was performed due to the observational design of the study. A 2-sided *P*-value <.05 was considered statistically significant. Analyses were performed using SAS version 9.4.

## RESULTS

**Figure 1.**
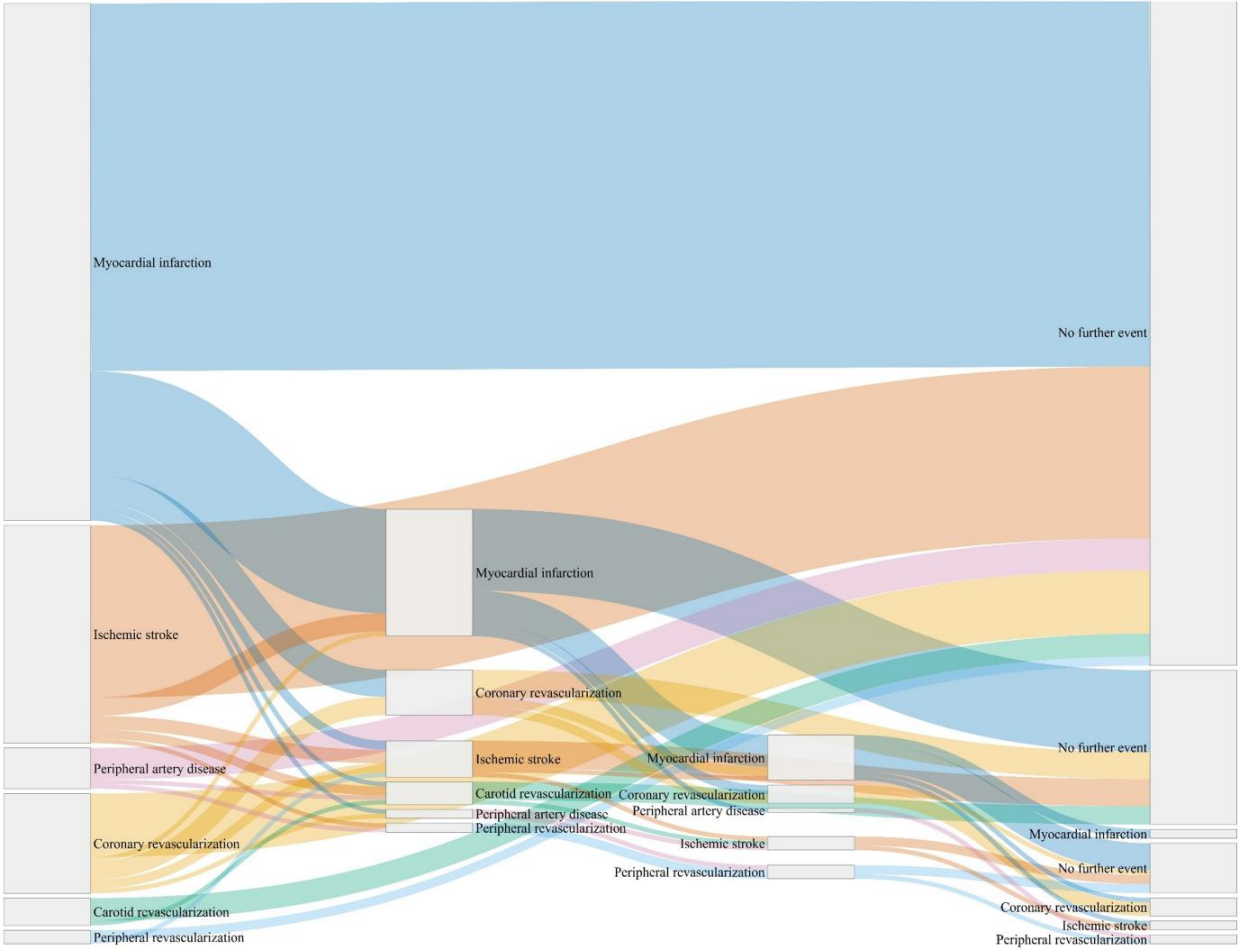
Trajectories of cardiovascular events after the first event among people with HIV. This Sankey diagram illustrates the sequence of cardiovascular events following the first cardiovascular event in people with HIV. The left column represents the type of first cardiovascular event, including myocardial infarction, ischemic stroke, peripheral artery disease, and coronary, carotid, or peripheral revascularization. Flows indicate transitions to subsequent cardiovascular outcomes after the first event, with follow-up censored at a maximum of 3 events.

Among 2955 PWH attending follow-up visits during the study period, 202 (6.8%) had established ASCVD. Of these 55 (27.2%) fulfilled the criteria for the newly defined extreme cardiovascular risk category: 14 (25.4%) with extensive multivessel arterial disease and 41 (74.5%) with recurrent vascular events. A total of 297 events were recorded: 157 myocardial infarctions, 45 coronary revascularizations or bypass procedures, 61 ischemic strokes, 12 symptomatic peripheral artery disease, 11 peripheral revascularizations, and 11 carotid revascularizations. Trajectories of cardiovascular events after the first event among PWH are depicted in Figure 1.

Table 1 summarizes and compares the clinical, laboratory, and demographic characteristics of PWH classified as very high versus extreme risk. Although most demographic and HIV-related characteristics were comparable between groups, PWH at extreme-risk were older (median age 67 [IQR 64–73] vs 64 [IQR 59–70] years, *P* = .001) and more frequently had a history of AIDS-defining events (41.8% vs 22.4%, *P* = .008). The prevalence of active smoking was high in both groups affecting nearly 55% of PWH. Use of combination lipid-lowering treatment (LLT) was limited (88/202, 43.6%), while advanced LDL-C–lowering agents such as PCSK9 inhibitors or bempedoic acid were rarely prescribed in both groups. Additionally, 9.4% of individuals did not receive any lipid-lowering prescription. A large proportion of PWH failed to achieve the guideline-recommended LDL-C targets: 79.6% and 72.7% had LDL-C levels above 55 mg/dL among PWH at very high and extreme cardiovascular risk, respectively. Only 10.9% of PWH at extreme cardiovascular risk achieved the guideline-recommended LDL-C target of <40 mg/dL.

**Table 1.** Characteristics of the Study Population

	Overall	Very High CVD Risk	Extreme CVD Risk	
	N = 202	n = 147 (72.8%)	n = 55 (27.2%)	*P*-value
**Age (years), median [IQR]**	65 [61, 70]	64 [59, 70]	67 [64, 73]	.001
**Female sex at birth, n (%)**	25 (12.4)	21 (14.3)	4 (7.3)	.233
**Mode of HIV acquisition, n (%)**				
Heterosexual	67 (33.2)	49 (33.3)	18 (32.7)	.038
MSM	50 (24.8)	42 (28.6)	8 (14.5)	
PWID	65 (32.2)	46 (31.3)	19 (34.5)	
Other/not specified	20 (9.9)	10 (6.8)	10 (18.2)	
**Time since HIV diagnosis (years), median [IQR]**	28 [21, 35]	28 [20, 35]	29 [24, 35]	.148
**Previous AIDS, n (%)**	56 (27.7)	33 (22.4)	23 (41.8)	.008
**Time on ART (years), median [IQR]**	24 [17, 29]	23 [15, 29]	26 [22, 29]	.038
**Previous or current abacavir use, n (%)**	105 (52.0)	75 (51)	30 (52.7)	.752
**CD4 nadir, median [IQR]**	178 [70, 282]	174 [73, 281]	185 [68, 283]	.738
**Current CD4 count, median [IQR]**	721 [485, 948]	732 [493, 972]	681 [478, 842]	.201
**HIV viral load <50 copies/mL, n (%)**	186 (92.1)	137 (93.2)	49 (89.1)	.382
**Comorbidities, n (%)**				
Hypertension	167 (82.7)	116 (78.9)	51 (92.7)	.022
Diabetes	47 (23.3)	31 (21.1)	16 (29.1)	.263
Chronic kidney disease				
ClCr <60 mL/min	56 (27.7)	40 (27.2)	16 (29.1)	.86
ClCr <30 mL/min	11 (5.4)	9 (6.1)	2 (3.6)	.731
Dialysis	4 (2.0)	3 (2.0)	1 (1.8)	.999
**Active smoking status, n (%)**	112 (55.4)	81 (55.1)	31 (56.4)	.999
**Lipid-lowering therapy, n (%)**				
Statin	88 (43.6)	66 (44.9)	22 (40.0)	.63
Ezetimibe	6 (3.0)	3 (2.0)	3 (5.5)	
Statin+ezetimibe	88 (43.6)	67 (45.6)	21 (38.2)	
Not specified	7 (3.5)	6 (4.1)	1 (1.8)	
None	19 (9.4)	17 (11.6)	2 (3.6)	
**New lipid-lowering agents, n (%)**				
Bempedoic acid	4 (2.0)	3 (2.0)	1 (1.8)	.999
Alirocumab	1 (0.5)	0 (0.0)	1 (1.8)	.272
Evolocumab	4 (2.0)	4 (2.7)	0 (0.0)	.576
**LDL-C cholesterol, median [IQR]**	71 [56, 105]	72 [58, 110]	70 [52, 89]	.200
<55 mg/dL, n (%)	45 (22.3)	30 (20.4)	15 (27.3)	0.343
<40 mg/dL, n (%)	15 (7.4)	9 (6.1)	6 (10.9)	0.243

Abbreviations: IQR, interquartile range; MSM, men who have sex with men; n, number; PWID, people who inject drugs.

## DISCUSSION

In this real-world cohort of PWH with established ASCVD, more than one quarter met criteria for the newly defined extreme cardiovascular risk category. This finding highlights a clinically important subgroup characterized by recurrent cardiovascular events and/or multivessel atherosclerotic disease. Although the ESC/EAS extreme-risk category has not yet been specifically validated in PWH and remains extrapolated from general population data, its application in this setting may help identify individuals requiring intensified secondary preventive intervention.

The higher prevalence of prior AIDS-defining events among PWH at extreme cardiovascular risk may reflect the long-term impact of prolonged or severe immune dysfunction on vascular health. Chronic immune activation, persistent inflammation, and cumulative immune damage have been implicated in accelerated atherosclerosis among PWH and may contribute to more extensive or recurrent ASCVD [1, 7], although causality cannot be inferred from this observational analysis.

For patients at very high and extreme ASCVD risk, clinical guidelines recommend early combination LLT to achieve stringent LDL-C targets, based on evidence from studies conducted in the general population [6]. Whether achieving these targets confers the same degree of cardiovascular protection in PWH, in whom persistent immune activation and inflammation may contribute to cardiovascular risk independently of LDL-C levels, remains to be specifically evaluated. Nevertheless, pursuing the most stringent LDL-C goals currently recommended by guidelines appears justified, given the excess cardiovascular risk in this population. However, despite the very high-risk profile of the study population, achievement of recommended LDL-C targets was poor. This is particularly concerning given the high burden of recurrent ASCVD in this setting. While the REPRIEVE trial highlighted important gaps and opportunities in primary prevention, our findings suggest that similar deficiencies persist even in the secondary prevention setting among patients at the highest levels of cardiovascular risk [4].

The poor achievement of LDL-C targets was likely related, at least in part, to limited use of combination lipid-lowering therapy and the rare prescription of advanced LDL-C–lowering agents, including PCSK9 inhibitors and bempedoic acid. These findings are consistent with previous reports, confirming that suboptimal lipid management and underutilization of guideline-recommended therapy represent a persistent and widespread challenge in the cardiovascular care of PWH [9-12]. Multiple barriers may contribute to this gap, including polypharmacy, concerns regarding drug–drug interactions, prescribing complexity, limited access to therapies, and clinical inertia. Fragmentation of cardiovascular care across HIV specialists, cardiologists, and primary care providers represents an additional structural barrier that may further exacerbate therapeutic inertia, leading to deferred or overlooked lipid management [13]. Addressing these barriers will require strategies at both the clinician and healthcare-system level, including simplified treatment algorithms, systematic cardiovascular risk reassessment, and improved patient education [14]. The persistently high prevalence of active smoking in our population further underscores the need for a comprehensive approach to cardiovascular prevention in PWH. In addition to lipid management, behavioral and modifiable cardiovascular risk factors, such as smoking habit, should be systematically addressed through targeted preventive interventions [15].

Overall, our observations highlight the need to improve cardiovascular risk awareness among healthcare professionals caring for PWH. As cardiovascular disease becomes an increasingly important contributor to morbidity and mortality in aging PWH, stricter implementation of cardiovascular prevention strategies should become a priority in routine HIV care. In this regard, clearer and uniform recommendations regarding cardiovascular risk assessment and lipid-lowering targets in PWH, supported by structured multidisciplinary collaboration among HIV specialists, cardiologists, and primary care providers, are needed to bridge the gap between guidelines and clinical practice.

This study has several limitations. Its retrospective, single-center design may limit generalizability. Adherence to LLT could not be systematically assessed and may have contributed to the low rates of LDL-C target achievement observed.

In conclusion, a substantial proportion of PWH with established ASCVD meet criteria for extreme cardiovascular risk; yet, achievement of guideline-recommended LDL-C targets remains markedly suboptimal. Bridging the gap between updated guideline recommendations and real-world clinical practice is essential to reduce the burden of recurrent ASCVD in this high-risk population.
